# Supplementary material for: Symptom-Based Dispatching in an Emergency Medical Communication Centre: Sensitivity, Specificity, and the Area under the ROC Curve
Source: Int J Environ Res Public Health. 2020 Nov 9;17(21):8254. doi: 10.3390/ijerph17218254 (PMC7664854; doi:10.3390/ijerph17218254)
Supplement: Supplementary file 1 [file ijerph-17-08254-s001.zip › Supplemental2.pdf]

Table S2: Symptoms epidemiology, dispatch priority, prevalence of severe symptoms, and association between dispatch / NACA score

	**All evaluations**, n (%)	**NACA score ≥ 4** (severe symptoms)	**Dispatch priority P1** (with L&S)	**Sensitivity** (95% CI)	**Specificity** (95% CI)	**PPV** (95%CI)	**NPV** (95%CI)
**All Symptoms evaluations**	**148'979**	**31'269 (21.0)**	**89'410 (60.0)**	**87.5 [87.1-87.8]**	**47.3 [47.0-47.6]**	**30.6 [30.3-30.9]**	**93.4 [93.2-93.6]**
**Age, years** (±SD)	58.9 (±26.3)	64.5 (±24.0)	56.2 (±26.6)				
**Male sex**	70'726 (47.5)	15'896 (22.5)	44'044 (62.3)				
**Female sex**	71'839 (48.2)	14'206 (19.8)	40'777 (56.8)				
**Heart, circulatory and respiratory**	**30'906 (20.7)**	**13'790 (44.6)**	**26'518 (85.8)**	**95.0 [94.6-95.3]**	**21.6 [21.0-22.2]**	**49.4 [48.9-50.0]**	**84.3 [83.1-85.3]**
Chest pain*	8'817 (5.9)	5'114 (58.0)	8'343 (94.6)	98.2 [97.8-98.5]	10.3 [9.3-11.3]	60.2 [59.1-61.2]	80.4 [76.5-83.9]
Cardiac arrest or death*	1'677 (1.1)	946 (56.4)	1'600 (95.4)	99.9 [99.4-100.0]	10.4 [8.3-12.8]	59.1 [56.6-61.5]	98.7 [93.0-100.0]
Dyspnoea / shortness of breath**	13'556 (9.1)	5'435 (40.1)	11'375 (83.9)	94.0 [93.3-94.6]	22.8 [21.9-23.7]	44.9 [44.0-45.8]	85.0 [83.4-86.4]
Heart rhythm disorder	3'357 (2.3)	1'002 (38.1)	2'704 (80.5)	8.9 [8.7-9.1]	25.3 [23.4-27.3]	45.4 [43.5-47.3]	76.6 [73.1-79.8]
Arterial hypotension, shock	1'303 (0.9)	407 (31.2)	949 (72.8)	89.4 [86.0-92.2]	34.7 [31.6-37.9]	38.4 [35.3-41.5]	87.9 [84.0-91.1]
Choking**	188 (0.1)	53 (28.2)	174 (92.6)	98.1 [89.9-100.0]	9.6 [5.2-15.9]	29.9 [23.2-37.3]	92.9 [66.1-99.8]
High blood pressure	1'388 (0.9)	349 (25.1)	890 (64.1)	79.9 [75.4-84.0]	41.2 [38.2-44.3]	31.3 [28.3-34.5]	85.9 [82.6-88.9]
Respiratory difficulty in children under 6 years	620 (0.4)	105 (16.9)	483 (77.9)	98.1 [93.3-99.8]	26.2 [22.5-30.2]	21.3 [17.8-25.3]	98.5 [94.8-99.8]
**Neurological, psychiatrics**	**37'658 (25.3)**	**9'036 (24.0)**	**22'919 (60.9)**	**86.1 [85.3-86.8]**	**47.1 [46.5-47.7]**	**33.9 [33.3-34.6]**	**91.5 [91.0-91.9]**
Stroke (or suspicion)	5'675 (3.8)	3'522 (62.1)	4'889 (86.1)	93.9 [93.1-94.7]	26.6 [24.7-28.5]	67.7 [66.3-69.0]	72.8 [69.5-75.9]
Coma / disturbance of consciousness*	5'213 (3.5)	1'949 (37.4)	4'909 (94.2)	97.1 [96.3-97.8]	7.6 [6.7-8.6]	38.6 [37.2-39.9]	81.6 [76.8-85.8]
Convulsions / seizure**	2'533 (1.7)	632 (25.0)	2'371 (93.6)	97.2 [95.5-98.3]	7.6 [6.4-8.9]	25.9 [24.1-27.7]	88.9 [83.0-93.3]
Headache*	1'295 (0.9)	316 (24.4)	765 (59.1)	77.5 [72.5-82.0]	46.9 [43.7-50.1]	32.0 [28.7-35.4]	86.6 [83.4-89.4]
Intoxication with drugs / overdose	3'483 (2.3)	804 (23.1)	2'388 (68.6)	83.3 [80.6-85.8]	35.9 [34.1-37.7]	28.1 [26.3-29.9]	87.8 [86.7-89.6]
Confusion / hallucination	2'470 (1.7)	331 (13.4)	530 (21.5)	44.7 [39.3-50.2]	82.1 [89.5-83.7]	27.9 [24.1-32.0]	90.6 [89.2-91.8]
Panic attack / suicidal ideation	2'802 (1.9)	344 (12.3)	717 (25.6)	49.4 [44.0-54.8]	77.7 [76.0-79.4]	23.7 [20.6-27.0]	91.6 [90.4-92.8]
Syncope / lipothymia	4'405 (3.0)	519 (11.8)	3'003 (68.2)	78.0 [74.2-81.5]	33.1 [31.7-34.6]	13.5 [12.3-14.8]	91.9 [90.3-93.2]
Seizure / febrile condition in children under 6 years	475 (0.3)	55 (11.6)	446 (93.9)	100.0 [93.5-100.0]	6.9 [4.7-9.8]	12.3 [9.4-15.7]	100.0 [88.1-100.0]
Agitation / aggressiveness	2'981 (2.0)	278 (9.3)	880 (29.5)	50.0 [44.0-56.0]	72.6 [71.0-74.3]	15.8 [13.4-18.4]	93.4 [92.2-94.4]
Alcoholic intoxication	4'421 (3.0)	209 (4.7)	1'901 (43.0)	53.6 [46.6-60.5]	57.5 [56.0-59.0]	5.9 [4.9-7.0]	96.2 [95.3-96.9]
Anxiety / depression	1'905 (1.3)	77 (4.0)	120 (6.3)	24.7 [15.6-35.8]	94.5 [93.3-95.5]	15.8 [9.8-23.6]	96.8 [95.8-97.5]
**General symptoms, other**	**32'452 (21.8)**	**4'934 (15.2)**	**16'470 (50.8)**	**79.1 [78.0-80.3]**	**54.3 [53.7-54.9]**	**23.7 [23.1-24.2]**	**93.6 [93.2-93.9]**
Allergies*	1'252 (0.8)	376 (30.0)	1058 (84.5)	95.2 [92.5-97.1]	20.1 [17.5-22.9]	33.8 [31.0-36.8]	90.7 [85.7-94.4]
New born and infant evaluation	109 (0.1)	27 (24.8)	109 (100.0)	100.0 [87.2-100.0]	0.0 [0.0-4.4]	24.8 [17.0-34.0]	0.0 [-]
Bleeding	2'888 (1.9)	691 (23.9)	1'700 (58.9)	84.8 [81.9-87.4]	49.3 [47.2-51.4]	34.5 [32.2-36.8]	91.1 [89.4-92.7]
Hypothermia	64 (0.0)	14 (21.9)	40 (62.5)	78.6 [49.2-95.3]	42.0 [28.2-56.8]	27.5 [14.6-43.9]	87.5 [67.6-97.3]
Person lying, without possibility to evaluate	7'289 (4.9)	1'558 (21.4)	6'351 (87.1)	96.5 [95.5-97.4]	15.4 [14.5-16.4]	23.8 [22.8-24.9]	94.2 [92.6-95.6]
Hypoglycaemia / hyperglycaemias	631 (0.4)	111 (17.6)	380 (60.2)	82.9 [74.6-89.4]	44.6 [40.3-49.0]	24.2 [20.0-28.8]	92.4 [88.4-95.4]
Unspecified malaise	7'023 (4.7)	946 (13.5)	3'977 (56.6)	73.2 [70.2-76.0]	45.9 [44.7-47.2]	17.4 [16.2-18.6]	91.7 [90.6-92.6]
Fever / flue-like condition	1'772 (1.2)	199 (11.2)	315 (17.8)	50.3 [43.1-57.4]	86.3 [84.5-88.0]	31.7 [26.6-37.2]	93.2 [91.8-94.4]
Other	9'117 (6.1)	883 (9.7)	2'351 (25.8)	56.3 [52.9-59.6]	77.5 [76.6-78.4]	21.1 [19.5-22.8]	94.3 [93.7-94.8]

Oto-rhino-laryngological problems	328 (0.2)	25 (7.6)	56 (17.1)	48.0 [27.8-68.7]	85.5 [81.0-89.2]	21.4 [11.6-34.4]	95.2 [92.0-97.4]
Ophthalmological problems	80 (0.1)	6 (7.5)	20 (25.0)	66.7 [22.3-95.7]	78.4 [67.3-87.1]	20.0 [5.7-43.7]	96.7 [88.5-99.6]
Pain / oedema of a limb	1'425 (0.8)	78 (5.5)	113 (7.9)	28.2 [18.6-39.5]	93.2 [91.8-94.5]	19.5 [12.6-28.0]	95.7 [94.5-96.8]
Social hospitalization	474 (0.3)	20 (4.2)	0 (0.0)	0.0 [0.0-16.9]	100.0 [99.2-100.0]	0.0 [-]	95.8 [93.6-97.4]
**Trauma, accidents**	**34'772 (23.3)**	**2'548 (7.3)**	**20'221 (58.2)**	**80.0 [78.4-81.6]**	**43.6 [43.0-44.1]**	**10.1 [9.7-10.5]**	**96.5 [96.2-96.8]**
Polytrauma (or suspicion)	73 (0.1)	42 (57.5)	73 (100.0)	100.0 [91.6-100.0]	0.0 [0.0-11.2]	57.5 [45.4-69.0]	0.0 [-]
Diving accident*	9 (0.0)	5 (55.6)	9 (100.0)	100.0 [47.8-100.0]	0.0 [0.0-60.2]	55.6 [21.2-86.3]	0.0 [-]
Electrocution	53 (0.0)	10 (18.9)	47 (88.7)	100 [69.2-100.0]	14.0 [5.3-27.9]	21.3 [10.7-35.7]	100.0 [54.1-100]
Burns	194 (0.1)	34 (17.5)	155 (79.9)	97.1 [84.7-99.9]	23.8 [17.4-31.1]	21.3 [15.1-28.6]	97.4 [86.5-99.9]
Ingestion, inhalation or exposure to a toxic	181 (0.1)	22 (12.2)	119 (65.7)	86.4 [65.1-97.1]	37.1 [29.6-45.1]	16.0 [9.9-23.8]	95.2 [86.5-99.0]
Chest trauma	974 (0.7)	118 (12.1)	587 (60.3)	77.1 [68.5-84.3]	42.1 [38.7-45.4]	15.5 [12.7-18.7]	93.0 [90.0-95.4]
Cranio-cerebral trauma	8'468 (5.7)	912 (10.8)	6'451 (76.2)	87.1 [84.7-89.2]	25.1 [24.2-26.1]	12.3 [11.5-13.1]	94.1 [93.0-95.1]
Bites	19 (0.0)	1 (9.5)	11 (57.9)	100.0 [2.5-100.0]	44.4 [21.5-69.2]	9.1 [0.2-41,3]	100.0 [63.1-100.0]
Abdominal or pelvic trauma	1'621 (1.1)	131 (8.1)	589 (36.3)	79.4 [71.4-86.0]	67.4 [65.0-69.8]	17.7 [14.7-21.0]	97.4 [96.2-98.3]
Wounds	2'151 (1.4)	162 (7.5)	1'134 (52.7)	82.1 [75.3-87.7]	49.7 [47.5-51.9]	11.7 [9.9-13.7]	97.1 [95.9-98.1]
Spinal trauma	2'632 (1.8)	158 (6.8)	1'559 (56.2)	80.3 [73.7-85.9]	42.3 [40.3-44.4]	9.2 [7.8-20.7]	96.7 [95.5-97.7]
Maxillofacial trauma	2'911 (2.0)	175 (6.0)	1'898 (65.2)	74.9 [67.8-81.1]	35.4 [33.6-37.2]	6.9 [5.8-8.1]	95.7 [94.2-96.8]
Trauma of a limb	15'486 (10.4)	758 (4.9)	7'589 (49.0)	70.3 [66.9-73.6]	52.1 [51.3-52.9]	7.0 [6.5-7.6]	97.2 [96.8-97.5]
**Abdominal, urological, gynaeco-obstetrical, back**	**13'191 (8.9)**	**961 (7.3)**	**3'282 (24.9)**	**55.0 [51.8-58.2]**	**77.5 [76.7-78.2]**	**16.1 [14.9-17.4]**	**95.6 [95.2-96.0]**
Threat of childbirth / childbirth*	370 (0.3)	105 (28.4)	336 (90.8)	98.1 [93.3-99.8]	12.1 [8.4-16.6]	30.7 [25.8-35.9]	94.1 [80.3-99.3]
Miscarriage, vaginal bleeding, pregnancy	365 (0.3)	100 (27.4)	244 (66.8)	83.0 [74.2-89.8]	39.2 [33.3-45.4]	34.0 [28.1-40.3]	86.0 [78.5-91.6]
Nausea, vomiting, diarrhoea	1'714 (1.2)	161 (9.4)	297 (17.3)	31.1 [24.0-38.8]	84.1 [82.2-85.9]	16.8 [12.8-21.6]	92.2 [90.6-93.5]
Abdominal pain (non-traumatic)*	6'571 (4.4)	447 (6.8)	1'950 (29.7)	56.4 [51.6-61.0]	72.3 [71.1-73.4]	12.9 [11.5-14.5]	95.8 [95.1-96.3]
Genital or urinary involvement	1'342 (0.9)	55 (4.1)	73 (5.4)	30.9 [19.1-44.8]	95.6 [94.4-96.7]	23.3 [14.1-34.6]	97.0 [95.9-97.9]
Back pain	2'075 (1.4)	68 (3.3)	182 (8.8)	22.1 [12.9-33.8]	91.7 [90.4-92.9]	8.2 [4.7-13.2]	97.2 [96.4-97.9]
Kidney pain	754 (0.5)	25 (3.3)	200 (26.5)	36.0 [18.0-57.5]	73.8 [70.4-77.0]	4.5 [2.1-8.4]	97.1 [95.4-98.3]

All variables given as numbers (group percentages in parenthesis) except age.Sensitivity, specificity, PPV, NPV, are shown in percentage

L&S: Light and Siren; CI: Confidence Interval; PPV: Positive Predictive Value; NPV: Negative Predictive Value. *: protocol exist; **: protocol exist since 01.01.2017
