# Supplementary figures and images for: Symptom-Based Dispatching in an Emergency Medical Communication Centre: Sensitivity, Specificity, and the Area under the ROC Curve
Source: Int J Environ Res Public Health. 2020 Nov 9;17(21):8254. doi: 10.3390/ijerph17218254 (PMC7664854; doi:10.3390/ijerph17218254)

**Figure S1:** Flow chart of study participants

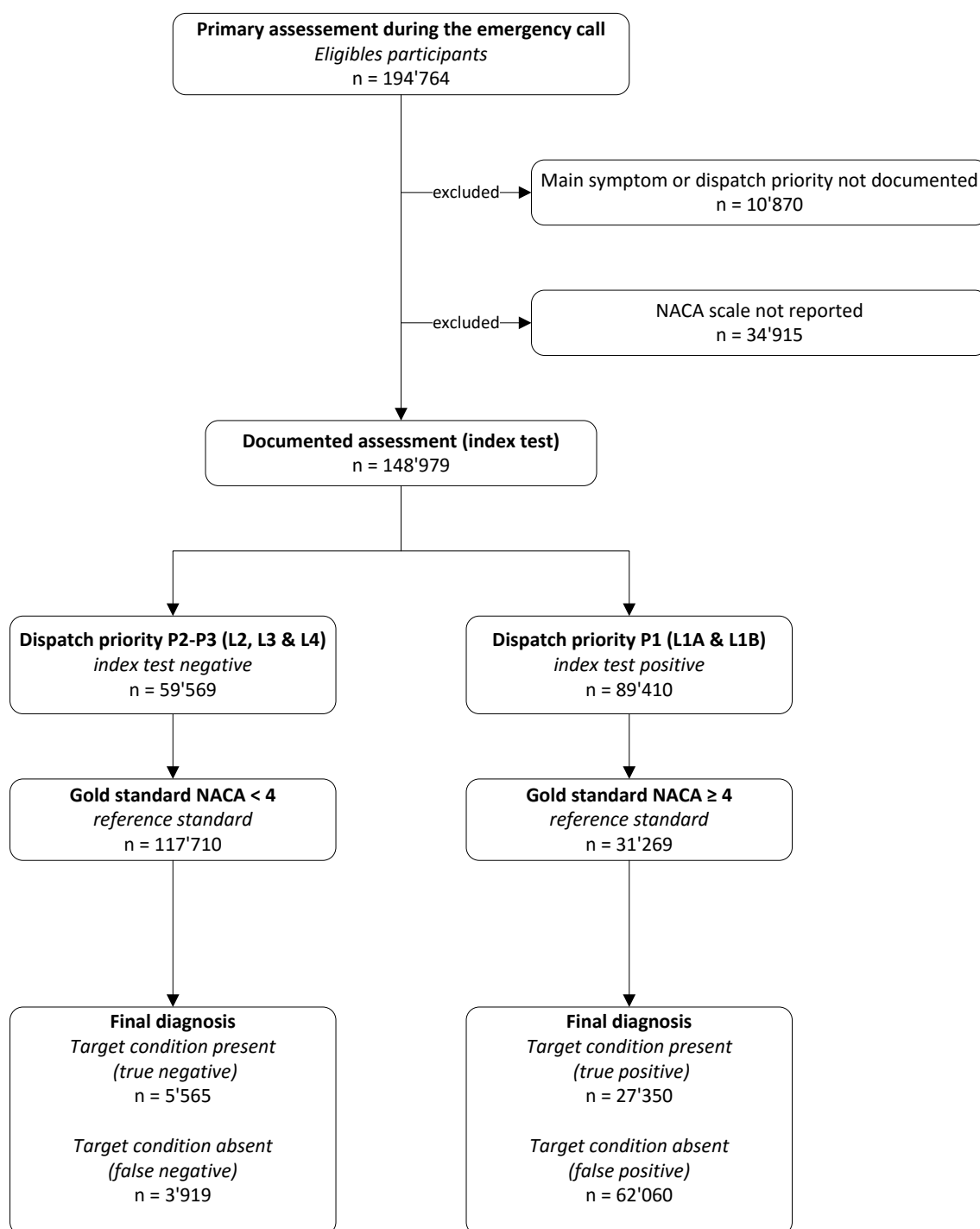

Supplement: Supplementary file 1 [file ijerph-17-08254-s001.zip › Supplemental1.pdf]
